# Effect of Cholesterol on the Structure and Composition of Glyco-DIBMA Lipid Particles

**DOI:** 10.1021/acs.langmuir.2c03019

**Published:** 2023-02-28

**Authors:** Julia Lenz, Andreas Haahr Larsen, Sandro Keller, Alessandra Luchini

**Affiliations:** † Molecular Biophysics, Technische Universität Kaiserslautern, Erwin-Schrödinger-Strasse 13, 67663 Kaiserslautern, Germany; ‡ Department of Neuroscience, University of Copenhagen, 2200 Copenhagen, Denmark; § Biophysics, Institute of Molecular Biosciences (IMB), NAWI Graz, University of Graz, Humboldtstrasse 50/III, 8010 Graz, Austria; ∥ Field of Excellence BioHealth, University of Graz, 8010 Graz, Austria; ⊥ BioTechMed-Graz, 8010 Graz, Austria; # European Spallation Source - ERIC, Partikel Gatan, Lund 224 84, Sweden; @ Department of Physics and Geology, University of Perugia, Via Alessandro Pascoli, 06123 Perugia, Italy

## Abstract

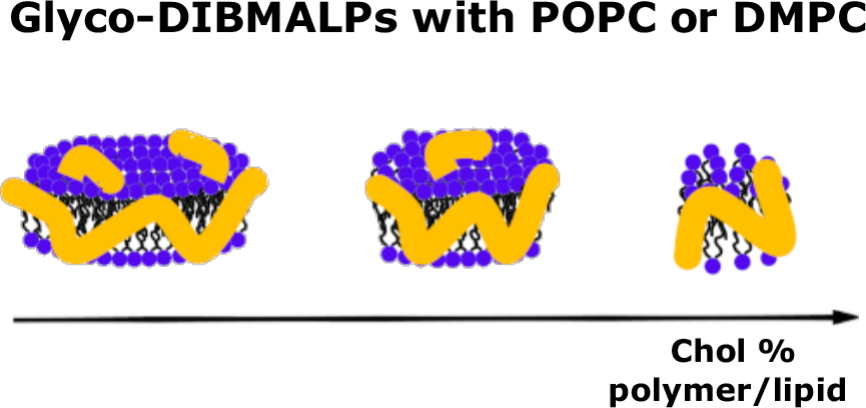

Different amphiphilic co-polymers have been introduced to produce polymer–lipid particles with nanodisc structure composed of an inner lipid bilayer and polymer chains self-assembled as an outer belt. These particles can be used to stabilize membrane proteins in solution and enable their characterization by means of biophysical methods, including small-angle X-ray scattering (SAXS). Some of these co-polymers have also been used to directly extract membrane proteins together with their associated lipids from native membranes. Styrene/maleic acid and diisobutylene/maleic acid are among the most commonly used co-polymers for producing polymer–lipid particles, named SMALPs and DIBMALPs, respectively. Recently, a new co-polymer, named Glyco-DIBMA, was produced by partial amidation of DIBMA with the amino sugar *N*-methyl-d-glucosamine. Polymer–lipid particles produced with Glyco-DIBMA, named Glyco-DIBMALPs, exhibit improved structural properties and stability compared to those of SMALPs and DIBMALPs while retaining the capability of directly extracting membrane proteins from native membranes. Here, we characterize the structure and lipid composition of Glyco-DIBMALPs produced with either 1-palmitoyl-2-oleoyl-*sn*-glycero-3-phosphocholine (POPC) or 1,2-dimyristoyl-*sn*-glycero-3-phosphocholine (DMPC). Glyco-DIBMALPs were also prepared with mixtures of either POPC or DMPC and cholesterol at different mole fractions. We estimated the lipid content in the Glyco-DIBMALPs and determined the particle structure and morphology by SAXS. We show that the Glyco-DIBMALPs are nanodisc-like particles whose size and shape depend on the polymer/lipid ratio. This is relevant for designing nanodisc particles with a tunable diameter according to the size of the membrane protein to be incorporated. We also report that the addition of >20 mol % cholesterol strongly perturbed the formation of Glyco-DIBMALPs. Altogether, we describe a detailed characterization of the Glyco-DIBMALPs, which provides relevant inputs for future application of these particles in the biophysical investigation of membrane proteins.

## Introduction

The structural and functional investigation of membrane proteins with conventional biochemical and biophysical methods often requires that the membrane protein molecules be stabilized in aqueous solutions.^1−3^ However, membrane proteins are found in cellular membranes, and the lipids enable proper biological function and stability. Therefore, different strategies for stabilizing membrane proteins in solution while keeping them associated with either synthetic or native lipids have been developed. Among these, incorporation of membrane proteins in nanodisc particles is a convenient approach for characterizing membrane proteins with a large variety of techniques, including X-ray and neutron scattering, nuclear magnetic resonance, and electron microscopy.^4−8^

Nanodiscs are 10–20 nm in size and are composed of a lipid bilayer core, in which the membrane protein of interest can be embedded. The lipid core is surrounded by a protein^5^ or polymer^9,10^ belt, which shields the hydrophobic region of the bilayer. A large variety of polymers have been suggested for the production of nanodiscs.^11,12^ Among the most commonly used are styrene/maleic acid (SMA),^9,10^ diisobutylene/maleic acid (DIBMA),^13,14^ and polymethacrylate (PMA) co-polymers.^11^ Other examples are zwitterionic and non-ionic polymers.^15,16^

Polymer–lipid particles composed of SMA and DIBMA, also known as SMALPs and DIBMALPs, respectively, have been used successfully for the stabilization of membrane proteins in synthetic lipid environments and for direct extraction of membrane proteins from their native cellular membrane.^14,17−19^ SMA was the first co-polymer introduced for successful extraction of membrane protein from the native membrane. However, the ultraviolet (UV) absorption associated with the styrene group prevents the quantification of the protein concentration by light absorption in the UV spectrum, which is an important aspect during sample preparation.^20^ In addition, the SMALPs are structurally unstable in the presence of divalent cations.^20,21^ DIBMA, on the contrary, lacks aromatic groups and therefore has reduced absorption bands in the UV spectrum and a larger content of negative charges, which improves the solubility at physiological ionic strength and in the presence of biologically relevant cations such as Mg^2+^ and Ca^2+^.^20,22^ However, the size distribution of DIBMALPs is broader than that of SMALPs, and DIBMALPs have a larger average size, which are not ideal properties for some structural investigations.^23^

Recently, a glycosylated co-polymer, named Glyco-DIBMA, has been obtained by partial amidation of DIBMA with the amino sugar *N*-methyl-d-glucosamine.^24^ Glyco-DIBMA shows improved properties with respect to the formation of discoidal polymer–lipid particles (Glyco-DIBMALPs) compared with those of DIBMALPs. Glyco-DIBMA retains solubility similar to that of DIBMA, a similar UV absorption spectrum,^24^ and a smaller amount of negative charge. Indeed, the electrostatic repulsion between the polymer chains has been suggested to induce the broad size distribution and larger size of DIBMALPs compared to those of SMALPs, Glyco-DIBMALPs, and other neutral polymers.^23,25−27^ As recently reported, Glyco-DIBMALPs are stable at physiological pH and ionic strength and in the presence of divalent cations. The formation of Glyco-DIBMALPs with different synthetic phospholipids was tested together with the application of this co-polymer for the direct extraction of membrane proteins from *Escherichia coli* cells.^24^

In this study, we aim to explore further the structural properties of Glyco-DIBMALPs by combining analytical methods with small-angle X-ray scattering (SAXS) to characterize the particle morphology as a function of the lipid composition. For this purpose, Glyco-DIBMALPs were produced with two different phospholipids, either 1-palmitoyl-2-oleoyl-*sn*-glycero-3-phosphocholine (POPC) or 1,2-dimyristoyl-*sn*-glycero-3-phosphocholine (DMPC). POPC and DMPC have been widely used for the production of various kinds of nanodiscs^11,28^ and are also among the lipids commonly used for the production of biological membrane models.^29^ Both phospholipids were used alone or in mixture with 10–30 mol % cholesterol.

Altogether, we provide a structural model for Glyco-DIBMALPs obtained from the analysis of SAXS data together with detailed information about the lipid composition of the polymer–lipid particles, i.e., total lipid concentration, the number of lipids per particle, and the ratio of either DMPC or POPC to cholesterol. We also highlight the challenges in producing Glyco-DIBMALPs containing cholesterol and how the effect of cholesterol is dependent on the kind of phospholipid used for sample preparation. This information is relevant for future applications of these kinds of polymer–lipid particles for the investigation of membrane proteins.

## Materials and Methods

### Chemicals

POPC (≥99% pure), DMPC (≥99% pure), and cholesterol (≥99% pure) were purchased from Avanti Polar Lipids, Inc. (Alabaster, AL), and used without further purification. Glyco-DIBMA was synthesized by Glycon Biochemicals (Luckenwalde, Germany) according to the protocol reported elsewhere.^24^ Chloroform (≥99.5% pure), perchloric acid (70%), ammonium molybdate tetrahydrate (≥99% pure), and ascorbic acid (99% pure) were purchased from Sigma-Aldrich. The cholesterol concentration was determined with the cholesterol Quantification Assay Kit purchased from Sigma-Aldrich. This is based on an enzymatic reaction that converts cholesterol into a product, which absorbs at 570 nm. A calibration curve is first measured with standard samples provided together with the kit, and from this calibration curve and the absorbance measured for the Glyco-DIBMA samples, the cholestorol concentration can be estimated. Further details about the cholesterol Quantification Assay Kit are reported by Sigma-Aldrich (https://www.sigmaaldrich.com/deepweb/assets/sigmaaldrich/product/documents/420/556/mak043bul.pdf).

### Sample Preparation

To prepare the Glyco-DIBMA stock solution, approximately 3 mL of a 20% solution of polymer powder (w/v) in ultrapure water was dialyzed at room temperature against 800 mL of buffer [50 mM Tris and 150 mM NaCl (pH 7.4)] for 24 h with buffer exchange after 16 h. This step was used to produce a a Glyco-DIBMA solution in physiological buffer. The pH value needs to be adjusted gently to prevent the polymer from precipitating. Then, the concentration of the solution was determined by a refractometer using a d*n*/dρ of 0.151 L/kg.^24^

The preparation of Glyco-DIBMALPs involves mixing of lipid vesicles with Glyco-DIBMA. Lipid vesicles were produced by mixing the phospholipid, i.e., either POPC or DMPC, and 0, 10, 20, or 30 mol % cholesterol in chloroform at a total lipid concentration of 5 mM. The solvent was removed under nitrogen flow, and the resulting film was rehydrated with 50 mM Tris and 150 mM NaCl (pH 7.4) and sonicated for at least 30 min to favor the solubilization of the lipid film and the formation of the vesicles, with a final lipid concentration of 5 mM. Subsequently, different volumes of a Glyco-DIBMA solution (69.8 mg/mL) were added to produce polymer/lipid ratios in the range 0.5–8 (w/w). The samples were incubated overnight under gentle agitation at 25 °C in the case of POPC and 37 °C in the case of DMPC. This step allowed the polymer to interact with the vesicles and form Glyco-DIBMALPs.

### Size Exclusion Chromatography

To purify the Glyco-DIBMALPs from other kinds of lipid and polymer aggregates, size exclusion chromatography (SEC) was performed using a Superdex 200 10/300 increase column and by injecting 0.5 mL of sample. The flow rate was set to 1 mL/min, and the buffer [50 mM Tris and 150 mM NaCl (pH 7.4)] was used as the eluent. Purified samples were collected in 0.5 mL fractions. Some of the samples containing cholesterol showed the presence of a precipitate after incubation of the lipid vesicles with the polymer. For those samples, centrifugation at 6000 rpm for 1 min was applied to separate the aggregates in solution from the precipitate. The supernatant was subsequently purified by SEC. The UV absorption of Glyco-DIBMA was monitored at 255 nm as this is the wavelength of highest absorption (Figure S1).

### Dynamic Light Scattering

Dynamic light scattering measurements were performed on a Brookhaven Instruments Corp. instrument using a laser wavelength of 632.8 nm at 25 °C and a scattering angle of 90°. The size of the particles (hydrodynamic diameter) was determined from the recorded autocorrelation functions using the second-order cumulant method analysis, implemented in the instrument software. Data were collected 24 h after sample preparation, with the sample stored at −20, 4, and 25 °C (Figure S2).

### Phosphate Analysis

The phospholipid concentration in the prepared samples was estimated by phosphate analysis.^30,31^ Therefore, 0.2 mL of the corresponding SEC fraction was used. The phospholipids in the samples were chemically digested by adding 0.4 mL of perchloric acid (<72%) to each of the 0.2 mL aliquots and by keeping the solutions at 180 °C for 2 h. Such treatment promoted the release of the phosphate group. The ammonium molybdate reagent (MR) was prepared by dissolving 2.2 g of (NH_4_)_6_Mo_7_O_24_·4H_2_O in a solution of 14.3% (v/v) H_2_SO_4_ and ultrapure water. Once the sample had cooled to room temperature, 4 mL of the MR solution was added to each of the samples together with 0.4 mL of a freshly prepared ascorbic acid solution [10% (w/w)]. Subsequently, the samples were stored at 80 °C for 10 min. This produced an organic complex with the free phosphate, which exhibited UV absorbance at 812 nm. Absorption at this wavelength was monitored for the treated samples as well as for standard solutions containing known amounts of disodium phosphate. The standard solutions were used to obtain a standard curve for the UV absorption as a function of the phosphate concentration, which was used to extract the phosphate content in the investigated samples.

### Small-Angle X-ray Scattering (SAXS)

All samples were prepared as described above, stored at −20 °C, and shipped on ice until they were measured at the B21 BioSAXS beamline at Diamond, Oxfordshire.^32^ The temperature for the measurements was set to at 10 °C to prevent sample aggregation. This temperature is also well below the melting temperature of DMPC, i.e., 25 °C. The experimental data were automatically reduced using the pipeline available at the beamline. Data were collected as separate frames and checked individually before averaging to identify potential sample radiation damage. The reduced one-dimensional data were rebinned logarithmically, from approximately 2500 to 200 points per data set.

**Figure 1:**
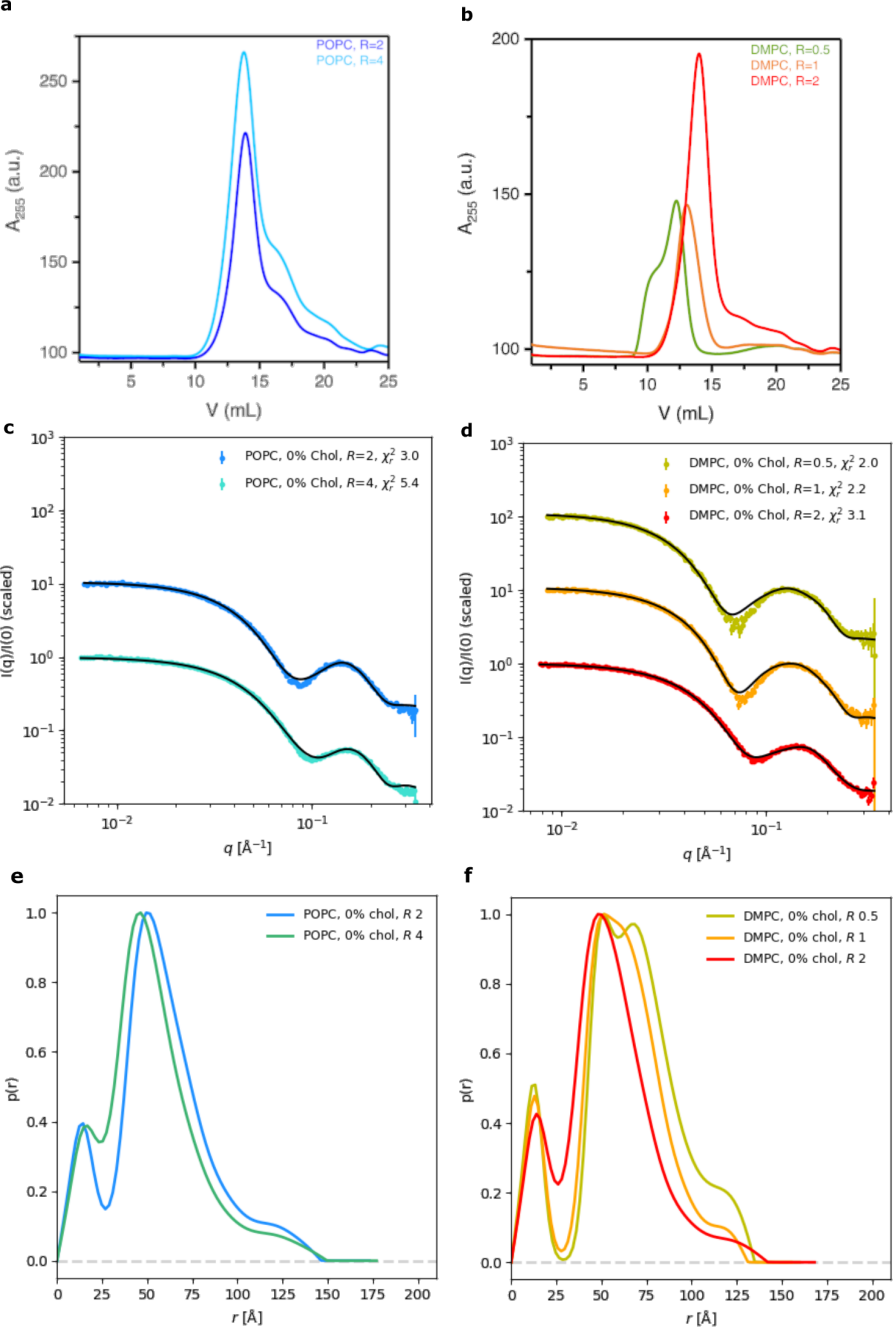
Glyco-DIBMALPs without cholesterol. (a) SEC data for Glyco-DIBMALPs with POPC at polymer/lipid ratios of 2 or 4. (b) SEC data for Glyco-DIBMALPs with DMPC at *R* values of 0.5, 1, or 2. (c) SAXS data for Glyco-DIBMALPs with POPC at different *R* values (colored error bars), fitted with an elliptical nanodisc model (black line). The reduced χ_r_^2^ values of the fits are displayed. (d) Corresponding SAXS data and fits for Glyco-DIBMALPs with DMPC. (e and f) *p*(*r*) functions for the SAXS data in panels c and d, respectively, normalized so the maximum value is unity. SAXS data were collected at 10 °C.

**Figure 2:**
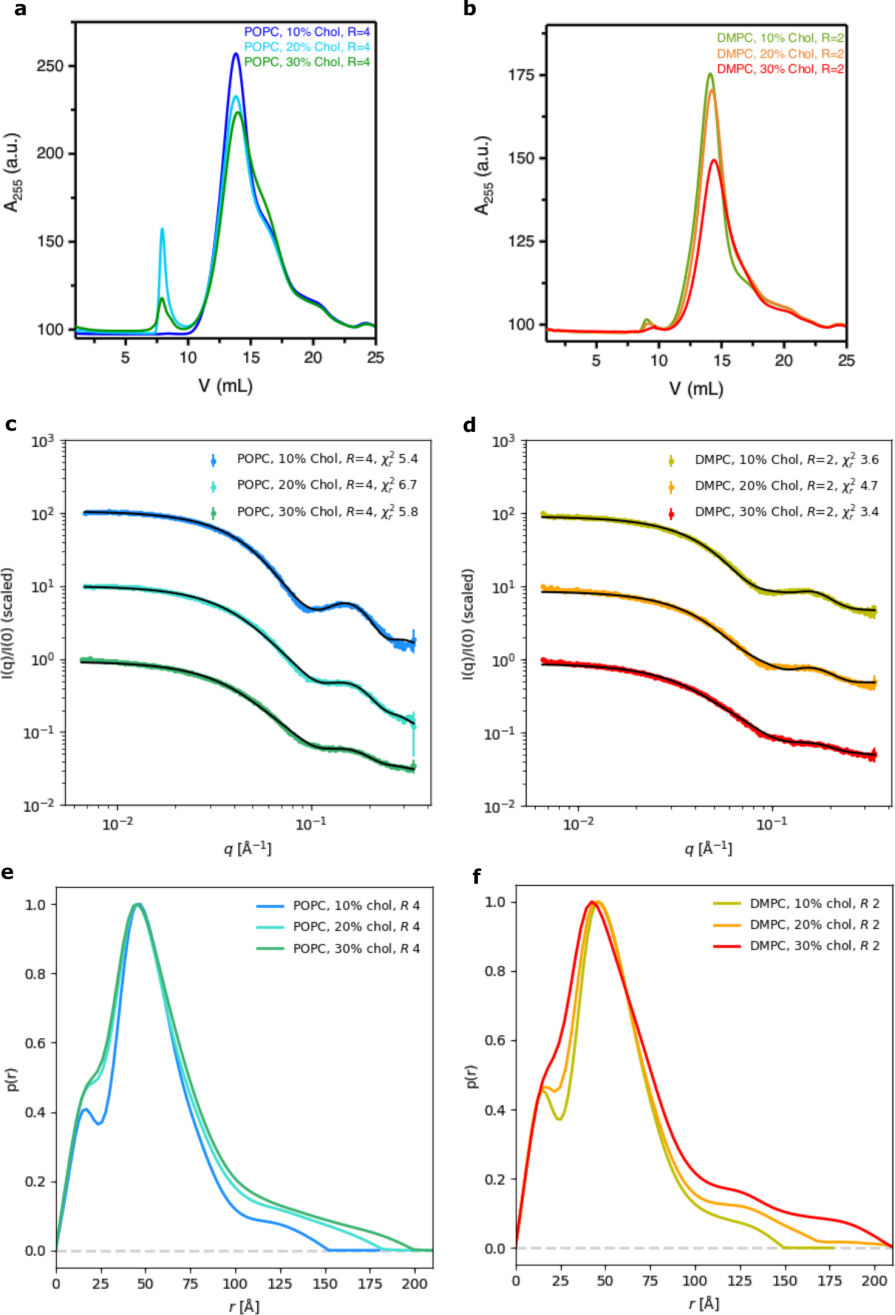
Glyco-DIBMALPs with cholesterol. (a) SEC data for Glyco-DIBMALP samples with POPC, a polymer/lipid ratio (*R*) of 4, and 10–30 mol % cholesterol. (b) Corresponding SEC data for Glyco-DIBMALPs with DMPC (*R* = 2). (c) SAXS data for Glyco-DIBMALPs with various amounts of cholesterol in the sample (colored dots), fitted with a model of elliptical nanodiscs (black line). χ_r_^2^ values of fits displayed. (d) Corresponding SAXS data for samples with DMPC. (e and f) *p*(*r*) functions for the SAXS data in panels c and d, respectively.

We used a Bayesian indirect Fourier transformation (BIFT)^33,34^ to assess the errors in data. The BIFT algorithm was accessed via BayesApp, version 1.0 (https://somo.chem.utk.edu/bayesapp/). A fit in the BIFT assumes only a smooth pair distance distribution, and though the fit to the data was good, as assessed by residuals and visual inspection, the resulting χ_r_^2^ values were much lower than expected for all data sets (∼0.1, compared to the expected value of unity). Using this, the errors were assessed to be overestimated by a factor of ∼3. Overestimated errors can lead to erroneous conclusions from the goodness of the fit (it is “too easy” to obtain a good fit), so the errors were rescaled accordingly. Rescaling did not affect the minimum found in the fitting process but provided better estimates of the goodness of fit as well as the uncertainty of the refined parameters. The method has been tested and described in detail previously.^34^ BIFT was also used to generate the pair distance distribution functions, *p*(*r*) (Figures 1e,f and 2e,f).

### Analysis of SAXS Data

#### Nanodisc Model

SAXS data were fitted with a model of discoidal particles with an adjustable number of polymer chains forming the amphiphilic belt, based on previous models for MSP-based^35,36^ and peptide-based nanodiscs.^37,38^ The model is based on stacked cylinders (Figure S3). An orientation-dependent radius of an elliptical cylinder with minor radius *r* and major radius *εr* is given as . The form factor amplitude of an elliptical cylinder depends on *r*_θ_ as well as the azimuthal angle (α) and the cylinder length (*L*):^39^
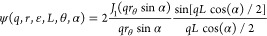
where *J*_1_(*x*) is the first-order Bessel function. An orientation-dependent intensity amplitude can then be constructed for a nanodisc as a sum of its constituents:
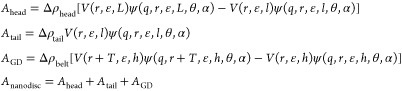
The form factor amplitudes are volume-weighted averages, where *V*(*r*, ε, *L*) = *πr*^2^*εL* is the volume of an elliptical disc with minor radius *r* and height *L*. Δρ_*i*_ terms are the excess scattering length densities for lipid headgroups (head), lipid tail groups (tail), and Glyco-DIBMA (GD). *r* is the minor radius of the bilayer. *T* is the thickness of the polymer belt. *L* is the height of the lipid bilayer. *l* is the height of its hydrophobic core (lipid tails), and *h* is the height of the polymer belt. The intensity is thus a function of the nine model parameters, **p** = {*r*, *T*, *l*, *L*, *h*, ε, Δρ_head_, Δρ_tail_, Δρ_GD_}, and the amplitude of the momentum transfer, which is given as *q* = 2π sin(θ)/λ, where 2θ is the scattering angle and λ is the wavelength. The interfaces of the actual particles are more rough than the geometrical model, which is taken into account by a surface roughness parameter, σ_*R*_.^36^ The model intensity is then calculated by integrating over all orientations, adjusting by the surface roughness, multiplying by a concentration-dependent scaling factor, *C*, and subtracting a constant background, *B*:
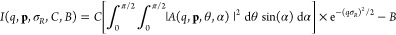


#### Constraining the Nanodisc Model

To reduce the degree of freedom in the model, we applied two constraints. First, the height of the Glyco-DIBMA belt, *h*, was constrained to take the same value as the height of hydrophobic core of the lipid bilayer, *l*, i.e., *l* = *h*. Second, from the EM data of SMALPs,^40,41^ we concluded that the polymer belt has an approximate hemispherical cross section. The radius of a hemisphere is *h*/2, so the cross-sectional area is *A*_hemisph_ = *πh*^2^/8. However, we model the rim with a rectangular cross section, with cross-sectional area *A*_rect_ = *hT*. These areas must be the same, which allows us to reduce the number of parameters by expressing *T* in terms of *h*: *T* = *πh*/8 (Figure S3).

#### Reparameterization

We reparameterized the model in terms of physical rather than geometrical parameters (Figure S3). Specifically, the model was expressed in terms of the number of lipids per nanodisc, *N*_lip_, the area per lipid headgroup, *A*_lip_, and the molecular volumes, *V*_tail_, *V*_head_, and *V*_GD_, using the equalities
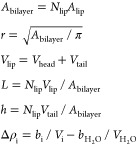
where the subscript *i* in the last equation denotes either head, tail, or GD. Some Glyco-DIBMA molecules could insert into the lipid bilayer. This was introduced into the model by a parameter, *n*_GD_, which is the number of Glyco-DIBMA molecules per lipid. We also accounted for cholesterol via the parameter *n*_chol_, which is the average number of cholesterol molecules per phospholipid. The effective scattering lengths were therefore
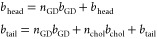
The molecular volumes were adjusted in the same way.

#### Fixing Parameters

The initial model had too many degrees of freedom to be sufficiently restrained by the SAXS data. This was assessed by the Bayesian indirect Fourier transformation, which gives as output a number of good parameters, which can be interpreted as the maximum number of parameters that can be refined from the data.^42^ The number of good parameters was ∼6 for each data set. This mismatch was mitigated by fixing parameters that were known *a priori*. Thus, the scattering lengths, *b*_i_, were calculated from the chemical formulas of the lipids and Glyco-DIBMA molecules. The number of cholesterols in the nanodiscs was measured in a separate assay, so *n*_chol_ could be fixed (Table S1). We also fixed the value of *A*_lip_ using literature values: 68.0 Å^2^ for POPC and 60.6 Å^2^ for DMPC.^43^*V*_tail_ and *V*_head_ were also fixed to previously reported values (Table 1), so the values of Δρ_head_ and Δρ_tail_ were refined only via *n*_GD_. An initial guess for *V*_GD_ was obtained from the molecular structure of the Glyco-DIBMA monomer and refined against the experimental data. Molecular volumes and scattering length densities are listed in Table 1. The remaining model parameters that were not fixed and therefore had to be refined against the SAXS data were *N*_lip_ (number of lipids per particle), ε, *V*_GD_ (molecular volume of Glyco-DIBMA), and *n*_GD_ (number of Glyco-DIBMAs per particle).

**Table 1:** Prior Values of Scattering Lengths and Molecular Volumes^a^

moiety	*V* (Å^2^)	chemical formula	Δρ (b_e_/Å^3^)
H_2_O	30	H_2_O	0.0
DM	791^38^	C_26_H_53_	–0.069
PO	927^35^	C_32_H_65_	–0.056
PC	319^35^	C_8_O_8_PNH_18_	0.143
cholesterol	678^44^	C_27_OH_46_	–0.015
Glyco-DIBMA	688^35^	C_20_O_8_NH_36_	–0.003

a The scattering lengths from the different molecules were calculated directly from their chemical formulas. *b*_e_ is the scattering length of an electron. Molecular volumes were estimated experimentally in previous studies.

Scaling, background, and roughness were fitted along with the remaining model parameters, resulting in a total of seven free parameters. Surface roughness was fitted globally; i.e., the same value was used for the fits to all data. This was done following a previously described protocol,^45^ by first fitting all data independently, then finding the average value of σ_*R*_, and refitting all data with σ_*R*_ fixed at this value. Thus, the number of free model parameters fitted to each data set was reduced to six, matching the number of good parameters^42^ (Table 2).

**Table 2:** Results from Bayesian Indirect Fourier Transformation, for Generation of the Pair Distance Distribution Function, *p*(*r*)^a^

sample	*D*_max_ (Å)	*R*_g_ (Å)	χ_r_^2^	*f*	*N*_g_	*N*_S_
POPC *R* = 2	143.1 ± 0.1	45.13 ± 0.05	0.08	0.29	6.5 ± 0.2	15.1
POPC *R* = 4	148.0 ± 0.9	41.71 ± 0.04	0.10	0.31	6.4 ± 0.2	15.7
DMPC *R* = 0.5	134.1 ± 1.0	50.41 ± 0.06	0.09	0.30	6.9 ± 0.2	14.1
DMPC *R* = 1	129.5 ± 0.8	45.69 ± 0.03	0.10	0.31	7.0 ± 0.1	13.6
DMPC *R* = 2	139.7 ± 1.0	41.93 ± 0.05	0.08	0.29	6.1 ± 0.2	14.8
POPC/cholesterol (90/10) *R* = 4	149.5 ± 0.6	42.39 ± 0.03	0.07	0.27	6.4 ± 0.1	15.8
POPC/cholesterol (80/20) *R* = 4	178 ± 2	47.6 ± 0.1	0.07	0.27	6.0 ± 0.1	18.8
POPC/cholesterol (70/30) *R* = 4	208 ± 5	52.4 ± 0.5	0.10	0.31	6.3 ± 0.2	22.2
DMPC/cholesterol (90/10) *R* = 2	148.5 ± 1.2	42.23 ± 0.08	0.08	0.28	5.5 ± 0.1	15.7
DMPC/cholesterol (80/20) *R* = 2	205 ± 3	50.5 ± 0.5	0.10	0.33	6.3 ± 0.2	21.6
DMPC/cholesterol (70/30) *R* = 2	214 ± 3	55.4 ± 0.4	0.09	0.30	5.4 ± 0.2	22.6

a *D*_max_ is the largest distance in the largest particle of the sample. *R*_g_ is the average radius of gyration. χ_r_^2^ is the reduced χ^2^ from the fit used to generate the *p*(*r*) function. *f* is the estimated correction factor for the experimental errors (σ_corrected_ = *fσ*). *N*_g_ is the estimated number of good parameters in the data. *N*_S_ is the number of Shannon channels, defined as *N*_S_ = *D*_max_(*q*_max_ – *q*_min_)/π.

#### Derived Parameters

For easy comparison and reporting, we defined an average radius, *r*_av_, as the average of the minor and major disc radii: *r*_av_ = [(*r* + *T*) + ε(*r* + *T*)]/2. Errors in this parameter was obtained by error propagation of the errors from the fitted parameters.

#### Assessment of Fits

The fits were assessed using visual inspection and the reduced χ^2^, which is given in terms of the measured intensities (*I*), the measured errors (σ), the calculated model intensity (*I*_mod_), which was evaluated at the measured momentum transfer values (*q*), the number of data points (*M*), and the number of free fitting parameters (*P*) in the model:
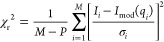


#### Implementation and Fitting Procedure

The model was implemented in Python3 using NumPy,^46^ and plotting was done with Matplotlib.^47^ Scripts for fitting and plotting are available at github.com/andreashlarsen/Lenz2022-DIBMALPs.

## Results and Discussion

### Glyco-DIBMALPs without Cholesterol

Glyco-DIBMALPs were prepared with either POPC or DMPC lipids following the protocol recently reported.^24^ The protocol consists of the incubation of lipid vesicles with Glyco-DIBMA followed by SEC purification to isolate the Glyco-DIBMALPs. Samples with Glyco-DIBMA and POPC were prepared with weight ratios (*R*) of 2 and 4. Ratios of 0.5, 1, and 2 were used for the samples with Glyco-DIBMA and DMPC. The polymer/lipid ratios were chosen on the basis of previously reported results, showing that when *R* < 2 for POPC or *R* < 0.5 for DMPC, the solubilization of the vesicles by Glyco-DIBMA was not effective.^24^

SEC analysis showed that POPC-containing samples with *R* values of 2 and 4 eluted in one main peak around 13.5 mL (Figure 1a). A fraction from the center of the peak was analyzed with dynamic light scattering and had an average size of 15 nm (Figure S4), which is in good agreement with previous results.^24^ The particle size distribution was not significantly affected by storage of the sample at 4 or −20 °C for 24 h (Figure S2). Broader peaks were observed for DMPC-containing samples with *R* values of 0.5 and 1 (Figure 1b). The peaks were also shifted toward smaller elution volumes, i.e., 10.5 and 12.5 mL, respectively. DMPC-containing samples with *R* values of 2, on the contrary, eluted at 13.5 mL, like the samples with POPC.

The SEC chromatograms reflected the Glyco-DIBMA concentration profile as a function of the elution volume. To check whether the eluting nanodiscs retained the original proportion of phospholipids, the phospholipid concentration was estimated by phosphate analysis for the fractions across the main peaks in the chromatograms. As a result, for both POPC and DMPC, the phosphate concentration showed a trend similar to that of the Glyco-DIBMA UV absorption (Figure S1).

SAXS data were collected to describe the structure of the Glyco-DIBMALPs (Figure 1c,d). All SAXS samples were collected from the center of the SEC elution peaks, as these fractions contained the highest Glyco-DIBMALP concentration.

Prior to model fitting, the data were transformed to provide the pair distance distribution function, *p*(*r*) (Figure 1e,f). The peak of the *p*(*r*) function for the POPC *R* = 2 sample was at larger sizes than the peak for the POPC *R* = 4 sample (Figure 1e). This is reflected in the radii of gyration, which were 45 Å for POPC *R* = 2 and 42 Å for POPC *R* = 4 (Table 2). Therefore, an increase in the polymer/lipid ratio results in the formation of smaller particles. The same pattern was observed for DMPC-containing particles. In this case, the radius of gyration decreased from 50 Å at *R* = 0.5 to 42 Å at *R* = 2, and a similar shift of the *p*(*r*) peak was observed (Figure 1f). The maximum size of the particles (*D*_max_), on the contrary, did not follow *R* systematically (Table 2), meaning that the size of the largest particles in the sample was not dictated by the polymer/lipid ratio. The *p*(*r*) functions also revealed some differences between samples prepared with POPC and DMPC. The samples with DMPC showed a more pronounced local minima at approximately 25 Å (compare panels e and f of Figure 1). A deeper minimum can be caused by a greater contrast between the inner lipid bilayer and the polymer belt, typically due to a larger bilayer.

The SAXS data from all samples without cholesterol could be fitted by a nanodisc model (Figure 1c,d). We also tested a core–shell micelle model against the SAXS data, but this model was less consistent with the data (Figure S5). The choice of the nanodisc model is also supported by previously reported negative-stain EM data.^24^ The nanodisc model includes a lipid bilayer at the center of the Glyco-DIBMALP, and the polymer chains are mainly localized at the rim of the bilayer, similar to what has previously been reported for MSP-based^35,36^ or peptide-based lipid nanodiscs.^37,38^ The polymer molecules thereby screen the hydrophobic region of the bilayer from the aqueous solvent. Although most of the polymers were at the rim of the nanodiscs, the model allowed for a fraction of polymers in the bilayer. According to our analysis, there were up to three Glyco-DIBMA chains for every 10 lipids in the nanodiscs (Table 3).

**Table 3:** Parameters and Goodness of Fit from SAXS Data Analysis^a^

sample	*r*_av_ (nm)	ε	*N*_lip_	*n*_GD_ (%)	χ_r_^2^	*V*_GD_
POPC *R* = 2	4.27 ± 0.05	1.86 ± 0.02	40.1 ± 0.6	17.3 ± 0.9	3.0	0.881 ± 0.002
POPC *R* = 4	3.49 ± 0.07	2.23 ± 0.02	17.0 ± 0.5	31.6 ± 0.8	5.4	0.901 ± 0.002
DMPC *R* = 0.5	5.06 ± 0.08	1.56 ± 0.02	96.6 ± 1.6	15.8 ± 0.8	2.0	0.862 ± 0.002
DMPC *R* = 1	4.50 ± 0.05	1.51 ± 0.01	74.9 ± 0.9	13.5 ± 0.7	2.2	0.854 ± 0.002
DMPC *R* = 2	3.83 ± 0.03	1.71 ± 0.01	42.8 ± 0.4	7.5 ± 0.6	3.1	0.821 ± 0.002
POPC/cholesterol (90/10), *R* = 4	3.43 ± 0.07	2.38 ± 0.02	13.8 ± 0.4	31.9 ± 0.6	5.4	0.915 ± 0.002
POPC/cholesterol (80/20), *R* = 4	3.04 ± 0.07	3.16 ± 0.02	5.6 ± 0.2	28.0 ± 0.6	6.7	0.946 ± 0.001
POPC/cholesterol (70/30), *R* = 4	3.20 ± 0.09	3.29 ± 0.02	5.9 ± 0.2	31.4 ± 0.8	5.8	0.944 ± 0.001
DMPC/cholesterol (90/10), *R* = 2	3.63 ± 0.03	1.87 ± 0.01	33.4 ± 0.4	1.8 ± 0.5	3.6	0.786 ± 0.002
DMPC/cholesterol (80/20), *R* = 2	3.67 ± 0.06	2.12 ± 0.02	28.7 ± 0.5	5.8 ± 0.8	4.7	0.787 ± 0.005
DMPC/cholesterol (70/30), *R* = 2	3.10 ± 0.13	3.64 ± 0.04	6.2 ± 0.3	31.4 ± 1.3	3.4	0.931 ± 0.003

a *r*_av_ is the averaged radius. ε is the ellipticity. *N*_lip_ is the number of lipids per Glyco-DIBMALPs. *n*_GD_ is the number of Glyco-DIBMA polymers per lipid in the bilayer of the nanodiscs. *V*_GD_ is the fitted value of the Glyco-DIBMA molecular volume relative to the expected value, i.e., 688 Å^3^ (Table 1). The derived geometrical parameters are listed in Table S2. SAXS data were collected at 10 °C.

The POPC *R* = 2 sample was best fitted with an elliptical nanodisc model with ellipticity of almost 2 and an average radius of 4.3 nm (Figure 1c), which is consistent with the hydrodynamic diameter measured by dynamic light scattering (Figure S2). The number of lipids per nanodisc was estimated to be 40. The POPC *R* = 4 sample was fitted with a smaller particle, i.e., with a smaller average radius (3.5 nm) and only 17 lipids per particle. These smaller particles also had a more pronounced ellipticity of >2. Increasing the polymer/lipid ratio thus results in the formation of smaller particles with fewer lipids (Table 3).

DMPC *R* = 0.5 Glyco-DIBMALPs had an average radius of approximately 5.1 nm and contained ∼97 lipids. DMPC *R* = 1 samples were better fitted with smaller nanodiscs, with a radius of 4.5 nm and 75 lipids per particles. Finally, the DMPC *R* = 2 sample was fitted with even smaller nanodiscs, with an average radius of 3.8 nm and 43 lipids per nanodisc. As the formation of Glyco-DIBMALPs with DMPC required a lower polymer/lipid ratio, the obtained particles were larger and contained more lipids than the Glyco-DIBMALPs with POPC. The DMPC-containing Glyco-DIBMA nanodiscs likewise had a less pronounced ellipticity (∼1.5) than the POPC-containing nanodiscs, to accommodate more lipids. Both of these aspects could contribute to the more pronounced minima in the *p*(*r*) functions (Figure 1e,f).

### Glyco-DIBMALPs with Cholesterol

Glyco-DIBMALPs were also prepared with either a mixture of POPC and cholesterol, i.e., POPC/cholesterol (90/10 mol/mol), POPC/cholesterol (80/20 mol/mol), and POPC/cholesterol (70/30 mol/mol), or DMPC and cholesterol, i.e., DMPC/cholesterol (90/10 mol/mol), DMPC/cholesterol (80/20 mol/mol), and DMPC/cholesterol (70/30 mol/mol). As cholesterol is known to affect the physicochemical properties of phospholipid bilayers, the purpose of these experiments was to quantify the content of cholesterol effectively loaded in the Glyco-DIBMALPs and investigate how it affects the particle structure.

In the case of POPC, lipid/polymer weight ratios of 2 and 4 were tested, as with these ratios Glyco-DIBMALPs with pure POPC were effectively formed. At *R* = 2, only POPC/cholesterol (90/10 mol/mol) was successfully prepared. At 20 mol % cholesterol, the formation of a precipitate was observed, and therefore, the polymer/lipid ratio was increased to 4 to improve the formation of the Glyco-DIBMALPs. An *R* of 4 was suitable for producing the samples with a POPC and cholesterol concentration of ≤20 mol %, but when the cholesterol concentration was further increased to 30 mol %, precipitation in the sample could not be avoided even by further increasing the polymer/lipid ratio. This sample was centrifuged, and the supernatant was used for characterization by SEC and SAXS.

For all of the POPC/cholesterol ratios tested, the SEC data exhibited the presence of a main peak at 13.5 mL (Figure 2a), like the POPC-containing samples without cholesterol. A second peak at a smaller elution volume was also observed. That could be related to the formation of other lipid–polymer aggregates than the Glyco-DIBMALPs. The lipid composition across the peak was also investigated by quantifying the POPC content (Figure S4). The fraction at the center of the peak was expected to contain the highest concentration of Glyco-DIBMALPs and was used to investigate the POPC/cholesterol ratio. In the case of the POPC/cholesterol mixture (90/10 mol/mol and 80/20 mol/mol), the actual lipid composition of this fraction agreed well with the nominal value, i.e., 8 mol % cholesterol and 18 mol % cholesterol, respectively (Table S1). On the contrary, for the POPC/cholesterol mixture (70/30 mol/mol), the actual cholesterol content of 15 mol % was well below the nominal value of 30 mol %. This observation, together with the precipitation in the POPC/cholesterol mixture (70/30), indicates that a concentration of cholesterol of >20 mol % is difficult to obtain in Glyco-DIBMALPs with POPC.

The behavior of the samples produced with mixtures of DMPC and cholesterol was substantially different from that of DMPC-containing samples without cholesterol. Sample precipitation occurred at all DMPC/cholesterol ratios, and increasing the polymer/lipid ratio did not mitigate this. Therefore, all samples were centrifuged, and the supernatant was used for sample characterization. The SEC data showed a profile similar to that of the data collected for POPC (Figure 2b), but the quantitative analysis of the lipids showed that the samples had a much lower phospholipid content compared to that of the samples prepared without cholesterol (Table S1). The measured DMPC/cholesterol ratios were only 1 mol % for DMPC/cholesterol (90/10), 1 mol % for DMPC/cholesterol (80/20), and 3 mol % for DMPC/cholesterol (70/30) and thus deviated substantially from the nominal composition. The cholesterol content was also measured in a fraction corresponding to the secondary peak at an elution volume of 9.5 mL (Figure 2b). This fraction had a cholesterol content of 26 mol %, suggesting that the cholesterol molecules concentrated into aggregates other than the Glyco-DIBMALPs. These could be vesicles or other kinds of aggregates with fewer polymer molecules, but we did not characterize these particles further. We concluded that it was difficult to load any substantial amount of cholesterol in Glyco-DIBMALPs prepared with DMPC.

The structure of the cholesterol-containing Glyco-DIBMALPs was also investigated with SAXS (Figure 2c,d). The *p*(*r*) functions show a less pronounced minimum, compared to those of samples without cholesterol (compare panels e and f of both Figures 1 and 2), which can be caused by a smaller bilayer. The *p*(*r*) functions generally have “tails” extending to larger values of *r*, when cholesterol is incorporated. In other words, the maximum distance, *D*_max_, of the sample increased upon addition of cholesterol (Table 2). This is likely due to a small fraction of larger particles being present in the sample, which is consistent with the observed precipitation.

The cholesterol-containing samples could also be fitted with the nanodisc model, despite the fits not being as good as the fits to the data without cholesterol, and some improvement could be achieved by adding a polydispersity parameter to the model (Table S2). Generally, incorporation of cholesterol resulted in smaller particles with fewer lipids and a higher ellipticity (Table 3). In particular, for the POPC/cholesterol (70/30), POPC/cholesterol (80/20), and DMPC/cholesterol (70/30) samples, the number of lipids per particle was <10, which could imply that a lipid bilayer is no longer present at the center of the particles, and the particles could be more homogeneously mixed polymer–lipid aggregates, rather than nanodisc-like Glyco-DIBMALPs. This is consistent with the lack of a minimum in the *p*(*r*) functions. The lipids, which were not part of the nanodisc-like particles, assembled in cholesterol-rich aggregates that were not characterized further.

### Discussion

**Figure 3:**
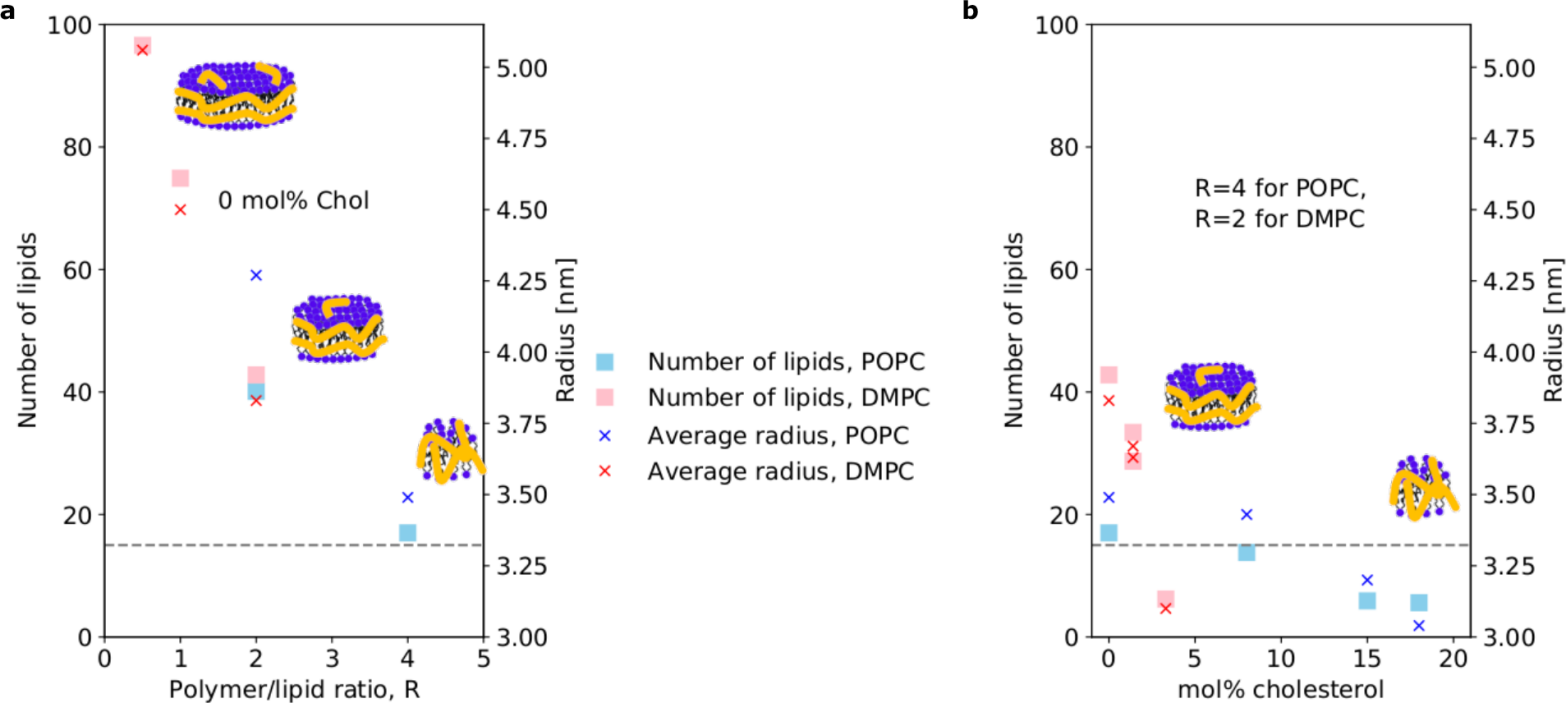
Particle size as a function of polymer/lipid ratio and cholesterol concentration. (a) The particle size and number of lipids decrease when the polymer/lipid ratio increases. (b) The particle size and number of lipids also decrease with the cholesterol concentration in the self-assembled particles. The average radius is the average of the major and minor axis of the elliptical nanodiscs. The approximate transition from discoidal particles to more globular particles without a proper lipid bilayer is indicated by the dotted line.

Our results are summarized in Figure 3. They show that nanodisc-like Glyco-DIBMALPs were formed from both pure POPC and pure DMPC. The polymer chains mainly form an outer belt around the lipid bilayer core. The averaged size of the particles is strongly dependent on the polymer/lipid ratio, with the Glyco-DIBMALPs prepared with DMPC and a polymer/lipid weight ratio of 0.5 being the largest and containing the most lipids per particle. The sizes decreased with an increase in the polymer/lipid ratio for both DMPC and POPC (Figure 3a). Furthermore, as also observed for another kind of nanodiscs,^35^ the Glyco-DIBMALPs exhibited an elliptical shape, which was also influenced by the polymer/lipid ratio, as the ellipticity decreases with an increase in the polymer/lipid ratio (Table 3). Similar results were also previously reported for nanodiscs with both polymer and protein belt.^13,24,48^

The addition of cholesterol to POPC or DMPC considerably affected the structure of the Glyco-DIBMALPs. In the case of POPC/cholesterol mixtures, we observed that the expected cholesterol amount could be loaded in the Glyco-DIBMALPs up to 20 mol %, although the particle average radius and number of lipids decreased significantly compared to those of the samples prepared with pure POPC (Figure 3b). At higher cholesterol concentrations, the sample was no longer stable, precipitation was observed, and the actual cholesterol concentration in the particle was substantially lower than the nominal value (Table S1). In the case of the DMPC/cholesterol mixtures, the cholesterol concentration in the particle was much lower than the nominal value, and precipitation during sample preparation was observed at all investigated DMPC/cholesterol ratios.

In the case of both POPC and DMPC, the elliptical nanodisc model described in the Materials and Methods and a circular nanodisc model were tested to analyze the collected SAXS data (Table S2). Ellipticity is entropically favorable and was observed for MSP-based nanodiscs;^35,45^ however, polydispersity is also expected for the Glyco-DIBMALPs, and the circular polydisperse nanodisc model was used previously for peptide-based nanodiscs.^37,38^ Albeit polydisperisity and ellipticity can be differentiated in SAXS data analysis,^49^ these parameters are usually not fitted simultaneously due to their correlation. Table S4 shows a comparison of the tested models: elliptical nanodiscs, circular nanodiscs, elliptical nanodiscs with polydispersity, and circular nanodiscs with polydispersity. The circular nanodisc model did not fit the data, as the symmetry of the model had to be broken. This can be introduced either by ellipticity or by polydispersity. The elliptical nanodisc model fitted best to the data corresponding to the Glyco-DIBMALPs prepared with either pure POPC or the mixtures of POPC and cholesterol. On the contrary, the data for Glyco-DIBMALPs with DMPC and DMPC/cholesterol were modeled equally well with the elliptical or polydisperse circular nanodisc model. Indeed, they were best fitted with the model of elliptical nanodiscs with polydispersity. However, in this case, we could not exclude the coexistence of different types of aggregates, some with nanodisc-like structure and some spherical aggregates with a more homogeneous distribution of polymer and lipid molecules (as suggested by the small number of lipids per particle reported in Table 3). Therefore, we did not go into further detail with modeling these data.

Glyco-DIBMALPs are formed by incubating lipid vesicles with a polymer solution. It has been reported for other amphiphilic co-polymers, such as SMA and DIBMA, that the polymer molecules initially associate with the vesicles and subsequently induce a destabilization of the lipid bilayer, which results in the extraction of lipid bilayer patches that form the lipid core of the nanodisc-like polymer–lipid particles.^50^ The packing of the lipids within the vesicle can affect the initial insertion of the polymer units within the hydrophobic region of the bilayer, which is a fundamental step for the formation of the discoidal particles.^51^ Although we did not specifically investigate the mechanism of formation of Glyco-DIBMALPs, on the basis of the similarities between Glyco-DIBMA and DIBMA with respect to producing polymer–lipid particles, we hypothesize that a similar mechanism could lead to the formation of Glyco-DIBMALPs. In addition, protein and polymer belt were reported to affect the physicochemical properties, e.g., melting temperature, of the bilayer within a nanodisc as compared to lipid vesicles.^48,52−54^ Altogether, the experiments reported here suggest that in general cholesterol can affect the formation of the polymer–lipid nanodiscs. POPC/cholesterol bilayers can be more efficiently extracted by Glyco-DIBMA and stabilized within a nanodisc than can DMPC/cholesterol bilayers. Nevertheless, also in the case of POPC, above 20 mol % cholesterol, the formation of the Glyco-DIBMALPs starts to be limited by the presence of cholesterol. This results in Glyco-DIBMA being able to extract only a few lipid molecules, i.e., <10, from the vesicles and forming aggregates with a higher concentration of polymer compared to the pure phospholipid Glyco-DIBMALPs. The different behaviors observed for the POPC/cholesterol and DMPC/cholesterol mixtures can be explained by the different packing of mixtures of saturated and monounsaturated phospholipids with cholesterol. Indeed, it has been well documented that cholesterol has a more pronounced condensing effect, i.e., decrease in the area per lipid and an increase in membrane thickness, in the case of a saturated phospholipid such as DMPC as compared to POPC.^55−57^ Such differences in the properties of the DMPC/cholesterol versus POPC/cholesterol bilayers could justify the formation of Glyco-DIBMALPs, with the process with the DMPC/cholesterol bilayer being more difficult. The formation of other kinds of nanodiscs, including protein, peptide, and polymer belts, with mixtures of phospholipids and cholesterol has been previously reported, although only a few studies investigated the actual amount of cholesterol loaded in the nanodiscs. Nanodiscs with POPC and the ApoA1 belt protein were produced with different cholesterol concentrations, and the effective loading of cholesterol was reported to be ≤20% mol.^58^ Peptide discs, nanodiscs with a belt composed of self-assembled 18A peptide molecules, were also produced with a mixture of POPC and cholesterol, and approximately 20% mol was the highest concentration that could be successfully used.^59^ Solubilization of a native membrane domain rich in cholesterol was also reported to be more difficult in the case of SMALPs. On the contrary, SMALPs with POPC and 30% mol cholesterol were used to study membrane proteins, although the cholesterol concentration within the SMALPs was not validated experimentally.^60^ Recently, mixtures of DOPC and cholesterol, with a high cholesterol concentration [30% (w/w)], were successfully used for the production of SMALPs.^61^ Altogether, the studies mentioned above indicate that the efficacy of cholesterol loading in nanodisc systems depends on several variables, including the concentration of cholesterol to be loaded, the belt length and composition, the nanodisc size, and the type of phospholipid used in combination with cholesterol. Often, high cholesterol concentrations (≥20–30 mol %) were found to negatively impact nanodisc formation, as also reported for the Glyco-DIBMALPs.

## Conclusions

Glyco-DIBMA is an amphiphilic co-polymer recently introduced as a promising candidate for producing polymer–lipid particles with a discoidal shape, i.e., Glyco-DIBMALPs, for the stabilization of membrane proteins in solution. Notably, DIBMA polymers can extract membrane proteins directly from native membranes, and the Glyco-DIBMA investigated here is an optimized version of DIBMA with a narrower size distribution. The formation of Glyco-DIBMALPs was initially tested with different synthetic phospholipids, and Glyco-DIBMA was also used for the extraction of membrane proteins from *E. coli* cells.^24^

In this work, we provide a structural characterization of Glyco-DIBMALPs, produced with either POPC or DMPC. Glyco-DIBMALPs were also prepared with mixtures of either POPC or DMPC and cholesterol at different mole percentages. The characterization focused on estimating the lipid content of the Glyco-DIBMALPs by analytical methods as well as determining the particle structure by SAXS. We show that, in the absence of cholesterol, Glyco-DIBMALPs produced from either POPC or DMPC exhibit a nanodisc structure with a variable diameter depending on the polymer/lipid ratio. On the contrary, in the presence of cholesterol, the Glyco-DIBMALPs produced from POPC or DMPC exhibited a different behavior. Glyco-DIBMALPs with POPC were able to efficiently incorporate cholesterol up to 20% mol, whereas Glyco-DIBMALPs made from DMPC included systematically lower cholesterol concentrations compared with the nominal composition of the sample. We ascribe the differences in the production of Glyco-DIBMALPs from DMPC versus POPC with high cholesterol concentrations to the different physicochemical properties of the POPC/cholesterol or DMPC/cholesterol bilayers compared to those of pure POPC and DMPC.

In summary, we report here a structural characterization of Glyco-DIBMALPs, which provides fundamental information and relevant inputs for future applications of these particles in the extraction of membrane proteins from native membranes and their stabilization in solution. Interestingly, the behavior of Glyco-DIBMA toward polymer/lipid particle formation was very different depending on the lipid mixture used (i.e., POPC/CHOL vs DMPC/CHOL), and a different polymer/lipid ratio was required to solubilize the lipids. Similar observations have previously been reported for other polymers and membrane–scaffold proteins forming nanodiscs. This suggests general features in the mechanism of formation of polymer- and protein-based nanodiscs, which deserve to be investigated more deeply.

## Supporting Information Available

UV spectrum of Glyco-DIBMA, additional SEC profiles, results of DLS measurements, further details about SAXS data analysis, and tables with the lipid concentrations estimated by phosphate analysis and cholesterol determination (PDF)
