# Supplementary material for: Effect of Cholesterol on the Structure and Composition of Glyco-DIBMA Lipid Particles
Source: Langmuir. 2023 Feb 28;39(10):3569–79. doi: 10.1021/acs.langmuir.2c03019 (PMC10018766; doi:10.1021/acs.langmuir.2c03019)
Supplement: Supplementary file 1 — la2c03019_si_001.pdf [file la2c03019_si_001.pdf]

Effect of cholesterol on the structure and composition of Glyco-DIBMA lipid particles
Supplementary Materials

Julia Lenz[1], Andreas Haahr Larsen[2], Sandro Keller[3], Alessandra Luchini[4,5*]

1 Molecular Biophysics, Technische Universität Kaiserslautern, Erwin-Schrödinger-Str. 13, 67663 Kaiserslautern, Germany

2 Department of Neuroscience, University of Copenhagen, 2200 Copenhagen, Denmark.

3 Biophysics, Institute of Molecular Biosciences (IMB), NAWI Graz, University of Graz, Humboldtstr. 50/III, 8010 Graz, Austria; Field of Excellence BioHealth, University of Graz, 8010 Graz, Austria; BioTechMed-Graz, 8010 Graz, Austria

4 European Spallation Source - ERIC, Partikelgatan, Lund, 224 84, Sweden;

5 current affiliation: Department of Physics and Geology, University of Perugia, via Alessandro Pascoli, 06123, Perugia, Italy

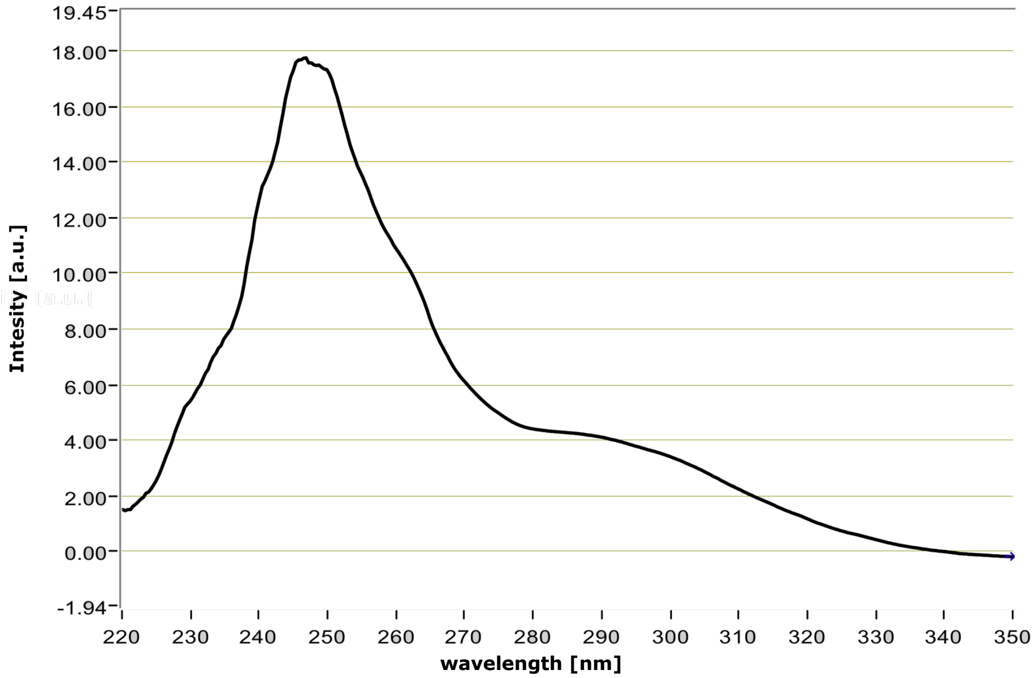

Figure S1: UV absorption spectrum collected for the Glyco-DIBMA solution used to produce the Glyco-DIBMALPs

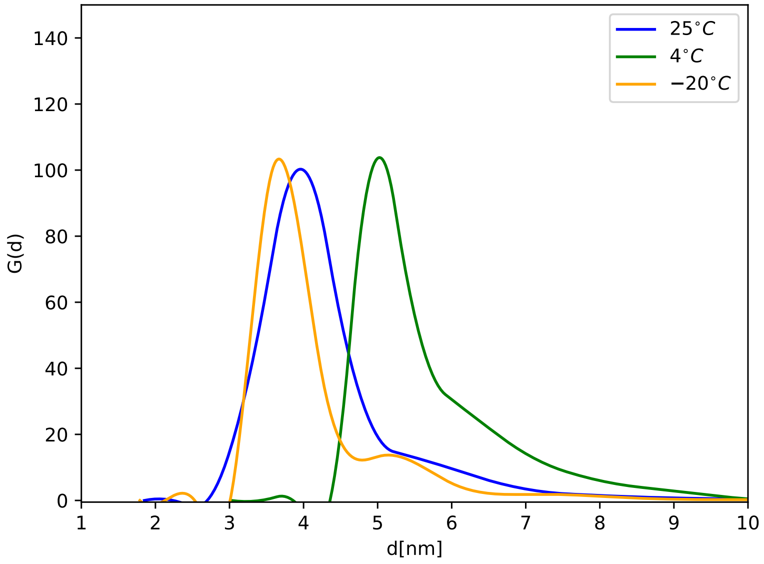

Figure S2: Scattered intensity distributions (G(d) of the hydrodynamic diameter. The distribution is normalised with the maximum equal to 100 and was calculated with the Brookhaven Instrument software. Data were collected at 25°C after 24h storage at 25°C, 4°C, and −20°C for the sample produced with POPC and polymer-lipid ration R=2.

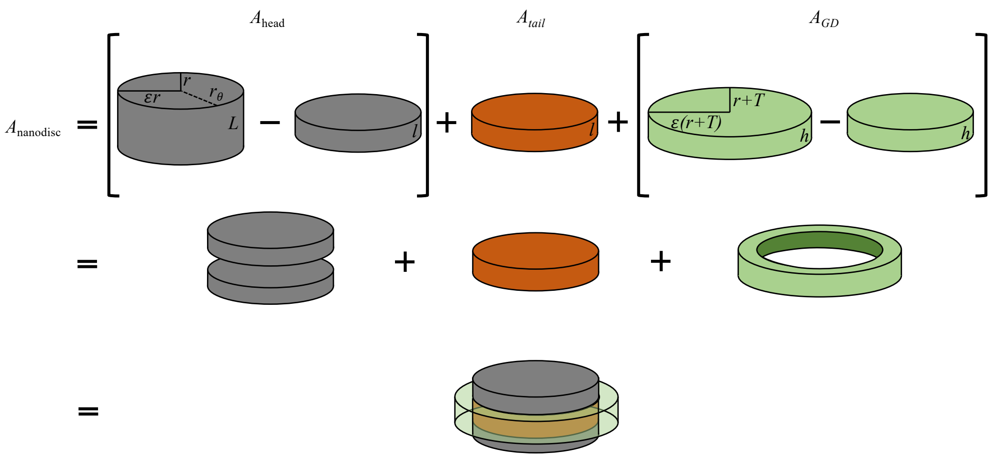

Figure S3: Schematic representation of the geometrical parameters within the nanodisc model for SAXS data analysis. h= height of the lipid bilayer within the nanodiscs, r=radius of the discoidal lipid bilayer, T=thickness of the polymer belt.

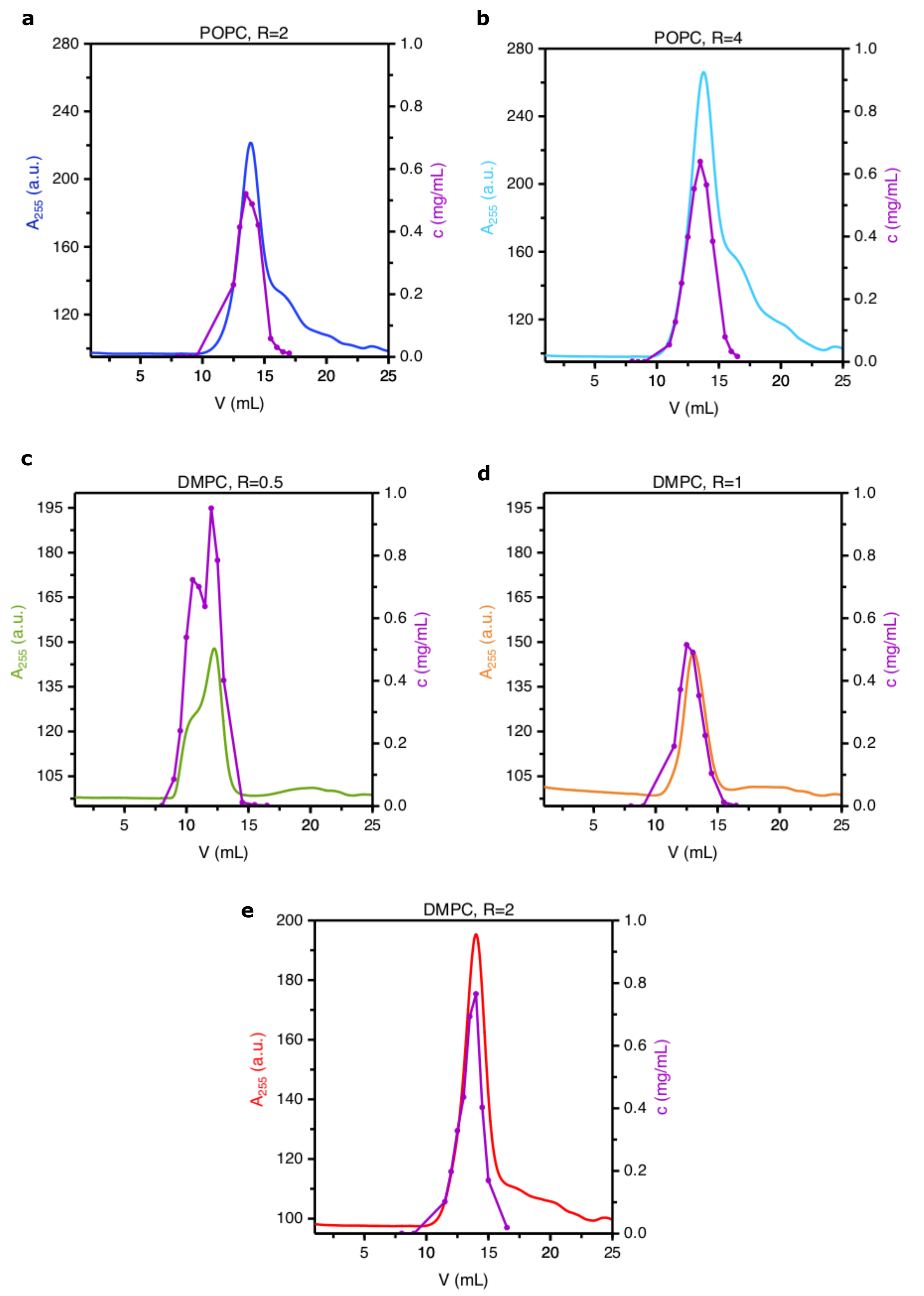

Figure S4: SEC chromatograms together with the corresponding lipid concentration profile for the Glyco-DIBMALPs prepared with POPC (a,b) and DMPC (c, d, e) at different polymer lipid ratios.

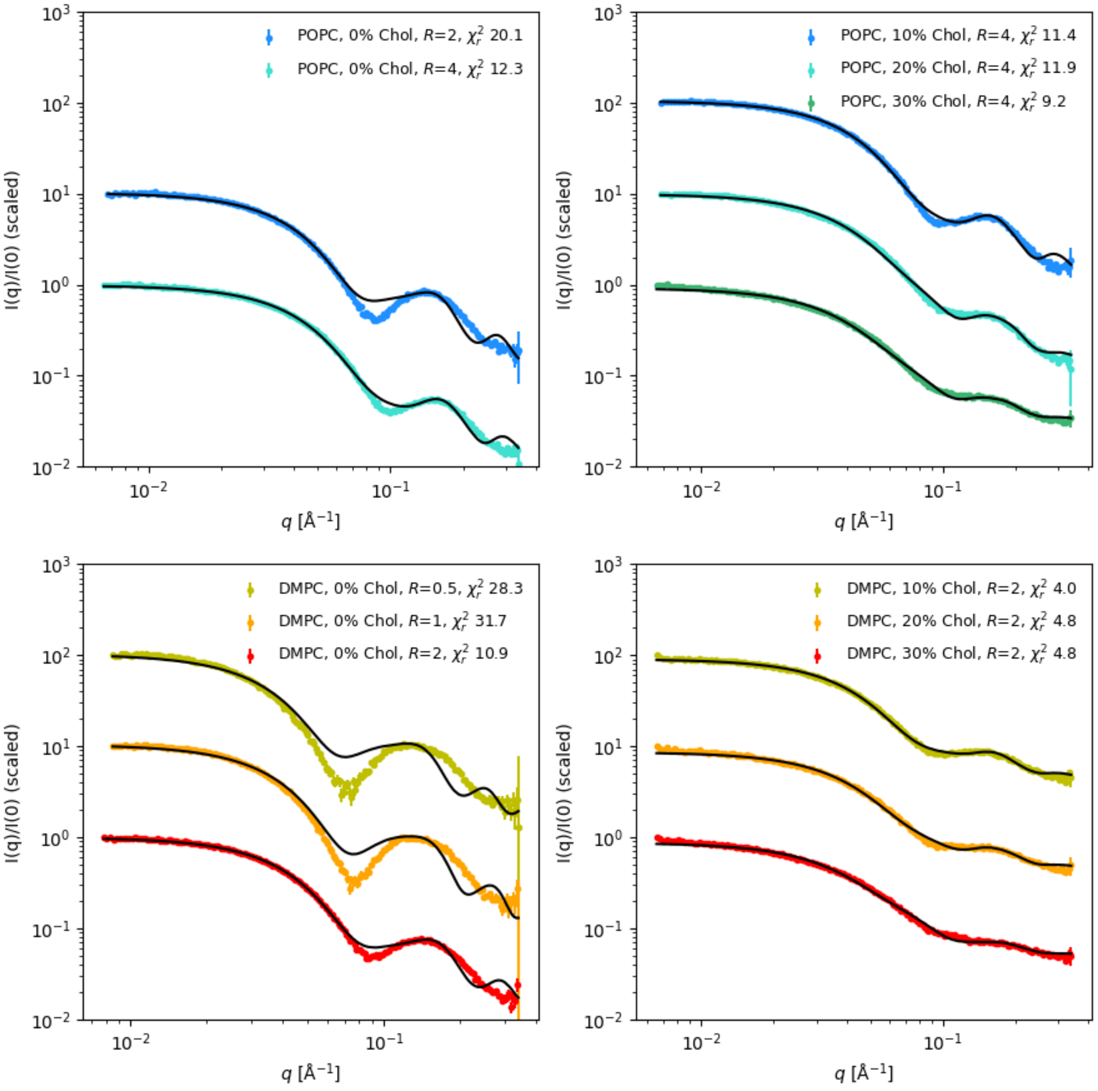

Figure S5: Glyco-DIBMALPs SAXS data fitted with a model of core-shell micelles. R is the polymer/lipid molar ratio. (a) POPC-based particles without Chol. (b) POPC-containing particles with cholesterol. (C) DMPC-containing particles without cholesterol. (d) DMPC-containing particles with cholesterol.

Sample	V [ml]	[PL] [mM]	[cholesterol] [mM]	PL/cholesterol ratio
POPC R=2	13.0	0.6987	-	-
POPC R=4	13.5	0.8410	-	-
DMPC R=0.5	12.0	0.6987	-	-
DMPC R=1	12.5	0.9590	-	-
DMPC R=2	13.5	0.9436	-	-
POPC/cholesterol (90/10) R=4	13.5	0.7545	0.0683	(92/8)
POPC/cholesterol (80/20) R=4	13.5	0.5763	0.1308	(82/18)
POPC/cholesterol (70/30) R=4	13.5	0.4244	0.0772	(85/15)
DMPC/cholesterol (90/10) R=2	14.0	0.6897	0.0098	(99/1)
DMPC/cholesterol (80/20) R=2	14.0	0.4891	0.0069	(99/1)
DMPC/cholesterol (70/30) R=2	14.0	0.3378	0.0115	(97/3)

Table S1: Phospholipid (PL) and cholesterol concentration. Nominal and measured values of molar ratios. V is the position of the fraction from the SEC, from which samples were taken for SAXS and concentration measurements.

Sample	r [Å]	h [Å]	L [Å]	T [Å]
POPC R=2	20.82	30.36	42.82	11.92
POPC R=4	13.58	33.04	48.18	12.97
DMPC R=0.5	30.53	29.20	42.81	12.97
DMPC R=1	30.53	29.20	42.81	11.47
DMPC R=2	26.87	28.72	41.86	11.28
POPC/cholesterol (90/10) R=4	12.23	34.78	50.06	13.66
POPC/cholesterol (80/20) R=4	7.80	36.23	50.97	14.23
POPC/cholesterol (70/30) R=4	8.00	36.26	51.64	14.24
DMPC/cholesterol (90/10) R=2	17.95	26.74	37.60	10.50
DMPC/cholesterol (80/20) R=2	16.65	27.46	39.03	10.78
DMPC/cholesterol (70/30) R=2	7.71	33.47	50.63	13.14

Table S2: Derived geometrical parameters (Figure S3).

Model	circ ND	ellip ND	pd circ ND	pd ellip ND
POPC R=2	22	3.0(ε=1.9)	4.8(pd=0.28)	2.5(ε=1.8, pd=0.17)
POPC R=4	60	5.4(ε=2.2)	13(pd=0.31)	5.0(ε=2.0, pd=0.18)
POPC/cholesterol (90/10) R=4	76	5.4(ε=2.2)	13(pd=0.31)	5.0(ε=2.0, pd=0.18)
POPC/cholesterol (80/20) R=4	85	6.7(ε=3.2)	20 (pd=0.32)	6.1(ε=2.6,pd=0.28)
POPC/cholesterol (70/30) R=4	59	5.8(ε=3.3)	14(pd=0.44)	5.2(ε=2.6, pd=0.30)
DMPC R=0.5	4.1	2.0(ε=1.6)	2.1(pd=0.20)	2.0(ε=1.6, pd=0.04)
DMPC R=1	5.9	2.2(ε=1.5)	2.3(pd=0.19)	2.2(ε=1.5,pd=0.8)
DMPC R=2	25	3.1 (ε=1.7)	2.8(pd=0.29)	1.8(ε=1.6,pd=0.23)
DMPC/cholesterol (90/10) R=2	25	3.6(ε=1.9)	3.1(pd=0.35)	1.7(ε=1.8, pd=0.29)
DMPC/cholesterol (80/20) R=2	19	4.7(ε=2.1)	4.7(pd=0.39)	2.7(ε=2.2, pd=0.31)
DMPC/cholesterol (70/30) R=2	26	3.4(ε=3.6)	5.3(pd=0.50)	2.8(ε=2.8, pd=0.36)

Table S3: Comparison of the χ_r^2 obtained by fitting the SAXS data with different models: circ ND: circular nanodiscs (monodisperse), ellip ND: elliptical nanodics (monodisperse), poly circ ND = polydisperse circular nanodiscs, pd ellip ND = polydisperse elliptical nanodiscs. ε indicate the ellipticity and *pd* is the relative polydisperisity. In case of Glyco-DIBMALPs with POPC, good fits are obtained with the elliptical nanodisc model, and only a small improvement is obtained by also including polydispersity on the nanodiscs size into the model. On the other hand, in case of Glyco-DIBMALPs with DMPC, fits of comparable quality are obtained by using the elliptical nanodisc model or the circular nanodisc with polydispersity. When cholesterol is added to POPC above 20% and for all the DMPC/cholesterol samples, the number of lipids per particle (see manuscript) is strongly decreased and the interpretation of the particle structure as more spherical and with lipid and polymer more mixed together is plausible.
